# Acute osteomyelitis, thrombophlebitis, and pulmonary embolism: a case report

**DOI:** 10.1186/s13256-023-04172-w

**Published:** 2023-10-28

**Authors:** Mohammad Sheikh Najeeb, Afif Alshwaiki, Nafiza Martini, Tamim Alsuliman, Banan Alkharat, Ali Alrstom

**Affiliations:** 1 Faculty of Medicine, Damascus University, Mashrou Dummar, Damascus, Syria; 2 Stemosis for Scientific Research, Damascus, Syria; 3 Hematology and Cell Therapy Department, Saint-Antoine Hospital, AP-HP Sorbonne University, Paris, France; 4 Infectious Disease, Internal Medicine Department, Al-Mouwasat (Damascus University Affiliated) Hospital, Damascus, Syria

**Keywords:** Acute osteomyelitis, Thrombophlebitis, Pulmonary embolism, MRI, Trauma

## Abstract

**Background:**

Septic pulmonary embolism (SPE), deep vein thrombophlebitis (DVT), and acute osteomyelitis (AOM) form a triad that is rarely seen in children and is usually associated with a history of trauma on long bones. Unfortunately, a delay in diagnosis is frequently observed in this syndrome, which places the patient at risk of life-threatening complications. This delay can largely be attributed to the failure to consider osteomyelitis as a potential underlying cause of DVT.

**Case presentation:**

In this case report, we present the case of a 16-year-old Arabian male who presented with limb trauma and fever. The patient had a delayed diagnosis of osteomyelitis, which resulted in the formation of an abscess and subsequent joint destruction. Surgical drainage and joint replacement surgery were deemed necessary for treatment.

**Conclusions:**

persistent fever along with a history of trauma on a long bone with signs of DVT of the limb in a child should raise concern for osteomyelitis and an MRI evaluation of the limb should be obtained.

## Introduction

Septic pulmonary embolism (SPE), deep vein thrombophlebitis (DVT), and acute osteomyelitis (AOM) form a triad that is rarely seen in children. Most cases have been associated with a history of trauma which presumably imposed patients to osteomyelitis that lead to thrombophlebitis and ultimately SPE. Other reported similar cases have been associated with *Staphylococcus aureus* (*S. aureus*) as a causal pathogen [1]. Herein, we report a rare case that associates Klebsiella pneumonia with this triad.

We are also willing to raise awareness about this syndrome as a possible cause of DVT in children and its unfortunate possible complications.

## Case presentation

**Fig. 1:**
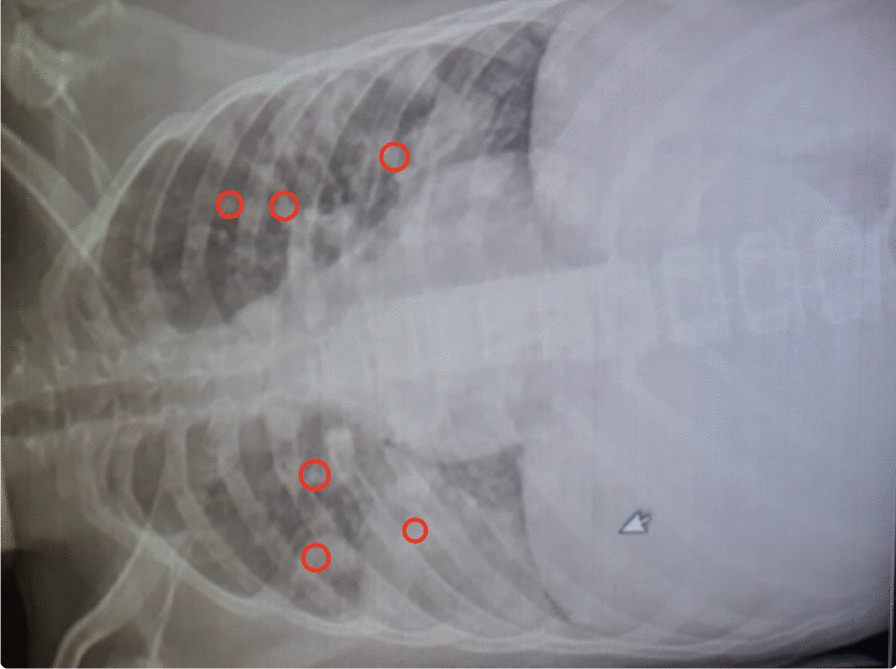
chest X-ray for the patient on admission showing bilateral pulmonary infiltrates (red circles). Arrow refer to bilateral pulmonary infiltrates

A 16-year-old Arabian male presented to our emergency department with a complaint of new-onset dyspnea and left hip pain with gait disturbance that started 1-week ago after a fall on his hip last week. The patient works as a car mechanic and his history included a recent urinary tract infection (UTI) with no further information regarding the clinical course and treatment of the condition. Hip X-ray was reported normal, and the Chest X-ray, which showed bilateral infiltrations, was presumed to be community-acquired pneumonia (Fig. 1). The patient was discharged on Levofloxacin. After 2-days the patient returned with worsening dyspnea, a 39 °C fever, and persistent left hip pain. The patient was admitted to our hospital. On admission, his respiratory rate was 24 breaths/minute, his SpO_2_ was 88% on ambient air with tachycardia (110 beats/minute).

Chest examination showed increased tactile fremitus in the right lung lower lobe, diffuse fine crackles in both lungs, and dullness to percussion on the right lung lower lobe. Left lower limb examination showed pan-edema, warm skin, and limited range of motion in both hip and knee joints.

Lab results showed respiratory failure with inflammatory response and elevated thrombotic markers. His hemoglobin was at 9.2 g/dL, white-cell count 35,000/µL, Neutrophils 87%, ESR 145 mm/hour, creatinine 2.1 mg/dL, Sodium 127 mmol/L, potassium 3.08 mmol/L, Ferritin 918 ng/dL, d-dimer 2850 ng/mL. Arterial blood gas indicated a pH of 7.52, SpO_2_ 89%, PO_2_ 54, PCO_2_ 28, and HCO3− of 21 mEq/L. Laboratory results are summarized in Table 1.

**Table 1:** A comparison table showing the patient’s laboratory values upon admission and 1 month later

Variable	Reference range	On admission	One month later
Hematocrit%	36.5–52.0	31.1	28
Hemoglobin, g/dL	12.0–17.4	9.2	8.3
White cell count	3.5–10.0	35	12.3
Neutrophils	40–70	87	77
Lymphocytes	22–44	9	16
Red cell count	4.00–5.50	3.45	3.25
MCV	27.0–32.0	70.5	84
Platelets count	150–400	180	1418
ESR, mm/hour	0–22	145	140
CRP, mg/L	0–5		134
Creatinine	0.6–1.4	2.1	0.15
Urea, mg/L	Aug-50	134	17
Sodium	135–145	127	137
Potassium	3.4–5.00	3.08	4.4
ALT, µ/L	Oct-55	41	172
Alkaline phos.	30–300		106
LDH WI	Less than 248		268
Ferritin	12–300	918	918
Iron	60–170		25
d-Dimer	Less than 500	2850	
pH	7.37–7.42	7.52	
CO_2_	35–45	28	

*LDH* Lactate dehydrogenase, *ALT* Alanine transaminase, *MCV* mean corpuscular volume, *ESR* Erythrocyte sedimentation rate, *CRP* C-reactive protein

**Fig. 2:**
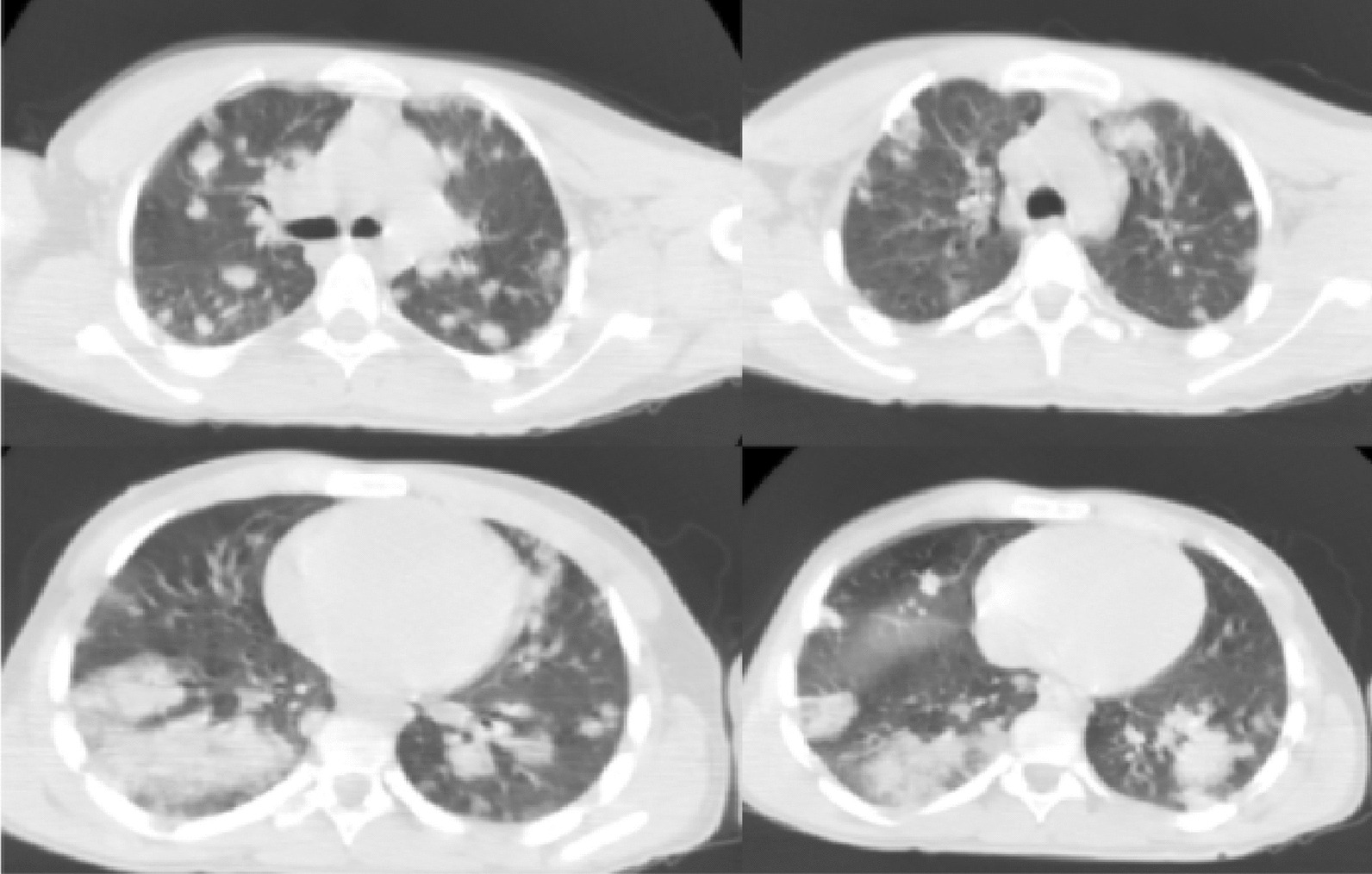
Computed tomography scan of the patient’s chest showing right lower lobe opacity with diffuse nodular opacities and cavitation in both lungs

Non-contrast chest CT revealed right lower lobe opacity and diffuse nodular opacities in both lungs (Fig. 2). Computed tomography pulmonary angiography (CT-PA) identified rounded nodular opacities in both lungs most of which are cavitary with the largest measuring 4 cm, with no evidence of massive pulmonary embolism.

Doppler ultrasound of the left lower limb shows Knee strain and left femoral vein thrombosis.

Blood cultures were drawn later after admission, and the patient was initially started on Enoxaparin and empiric antibiotic treatment with Ceftriaxone, Vancomycin, and Levofloxacin. Later, Blood cultures, along with bronchoalveolar lavage fluid cultures grew fluoroquinolone-sensitive extended-spectrum beta-lactamase (ESBL) Klebsiella pneumonia. All antibiotics were discontinued and meropenem was started.

The patient’s limb clinical symptoms were consistent with deep vein thrombosis (DVT), which was confirmed by ultrasound findings. Additionally, bilateral pneumonia with a confirmed cavitary lesion and suspected emboli were observed on CT-PA. The presence of positive blood and bronchoalveolar lavage fluid culture results further suggested the presence of septic pulmonary embolism (SPE).

Further evaluation through transesophageal echocardiography revealed a normal heart and valves with no vegetation. Based on these findings, the diagnosis of SPE associated with DVT caused by Klebsiella pneumonia was made.

After 2 weeks of treatment with meropenem, the patient’s respiratory status remarkably improved but he remained persistently febrile.

In light of these findings, an abscess was suspected, and a complete re-evaluation was started.

Laboratory investigation showed minimal improvement with almost similar values to the ones on admission (Table 1).

**Fig. 3:**
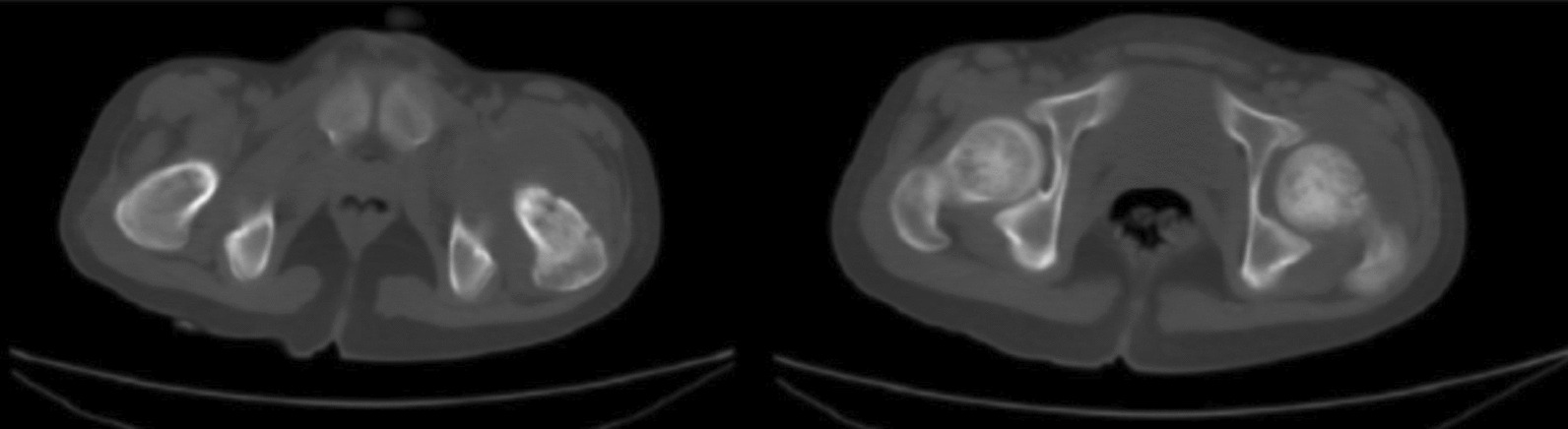
Left hip joint computed tomography showing a hypodense transverse lesion with a non-regular border on the intertrochanteric line that extends to the hip joint, indicating a fracture

Whole body scanning computed tomography was performed. Chest CT showed remarkable regression of lung opacities compared with the previous one on admission. Left hip joint CT showed a hypodense transverse lesion with a non-regular border on the intertrochanteric line that extends to the hip joint, indicating a fracture (Fig. 3). In addition, there was rich loculated effusion inside the joint that is suspicious of an abscess.

**Fig. 4:**
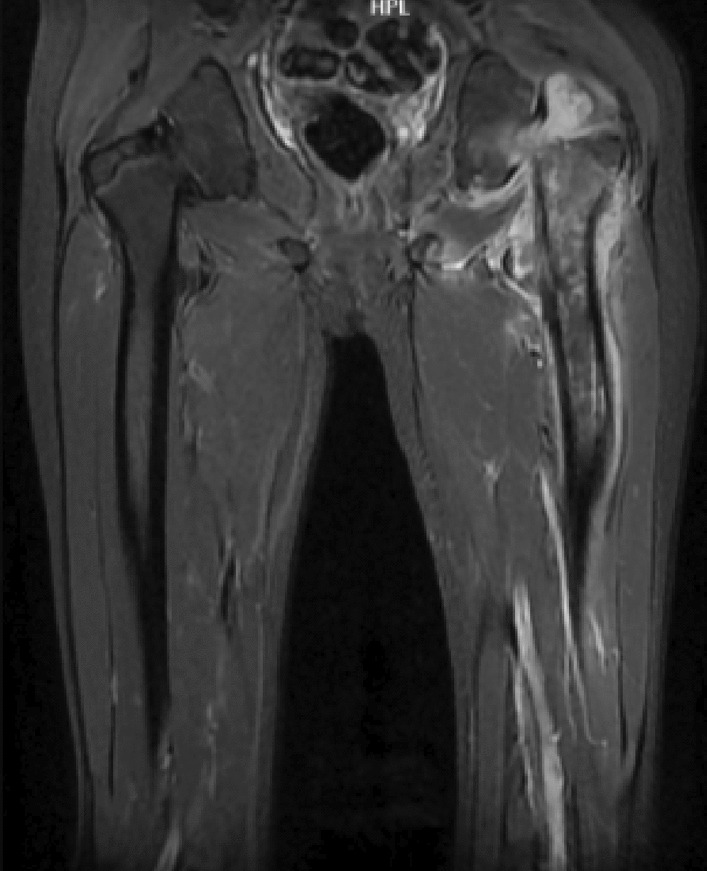
Magnetic Resonance Imaging of the hip joint showing bone marrow edema in the femur bone with joint effusion

Contrast MRI of the left hip joint showed traumatic bone marrow edema in the neck and body of the femur and a 13–18 mm loculated effusion in the joint that extended to the medial and lateral parts of the femur body with an extended joint capsule (Fig. 4). MRI and CT findings were highly suggestive for osteomyelitis with subsequent abscess formation.

The patient underwent an exploratory operation for the left hip joint, during which an abscess was detected, consequent drainage resulted in abundant pus exudate, and cultures grew fluoroquinolone-sensitive ESBL Klebsiella pneumonia. In addition, a fracture of the anterolateral part of the femoral neck was confirmed.

The patient was discharged with a prescription of oral Ciprofloxacin and IV Cefoperazone-Sulbactam for 4 weeks as blood and joint drainage fluid cultures grew fluoroquinolone-sensitive ESBL *Klebsiella pneumoniae*. Additionally, the patient was given non-weight-bearing instructions and physical therapy for the left hip joint. After discharge, the patient was examined on multiple occasions, and the lab workup showed normal ESR and CRP levels. However, the clinical examination of the hip joint showed joint stiffness and reduced mobility. As a result, the patient was referred to orthopedic specialists and underwent hip joint replacement surgery.

## Discussion

Osteomyelitis, thrombophlebitis, and septic pulmonary embolism are life-threatening triads usually present in young children.

Our patient’s history of minor trauma to his hip joint matches with LePage *et al.*’s findings, which stated that this syndrome typically happens after a history of minor trauma to long bones of lower extremities [1]. Although they emphasized the unclear sequential order in which the components of this triad develop [1], our patient history of UTI followed by hip trauma supports the hematogenous spread osteomyelitis theory [2].

While we think that the recent UTI may have played a role in the pathogenesis of the disease, we do not think that the three parts of the triad developed independently and then coincidentally converged. Instead, we propose a possible explanation: during childhood, there is a high blood flow to the metaphysis of the bones, particularly in the long bones. When combined with limb trauma, this can increase the risk of bone infection in cases of bacteremia, especially if the infecting organism has high virulence factors like Klebsiella pneumonia. As the disease was not detected early, the infection spread to the draining venous system of the limb, leading to DVT. The DVT then starts sending septic emboli to the lungs, causing cavitary pneumonia. This timeline is consistent with the patient’s initial complaint of leg pain, followed by the development of pneumonia symptoms a week later.

Several studies demonstrate the strong correlation between this triad and *Staphylococcus aureus* [1, 3, 4]. In our case, however, synovial fluid of the hip joint, blood, and bronchoalveolar fluid culture yielded Klebsiella pneumonia as a causal pathogen. This was Supported by the chest CT findings (Fig. 1), which reveal opacity in the right lower lobe with diffuse nodular opacities and cavitation in both lungs, which are common findings for Klebsiella pneumonia infection [5].

Diagnosis of this syndrome on the first presentation may be challenging for clinicians. Symptoms including pain and swelling of the limb with possible redness strongly suggest DVT.

Given the fact that the duplex ultrasound is the standard way to diagnose DVT, osteomyelitis may go unnoticed which misleads the diagnosis and hides the underlying etiology of the DVT. Thus, persistent fever along with a history of trauma on a long bone with signs of DVT of the limb in a child should raise concern for osteomyelitis and an MRI evaluation of the limb should be obtained.

Delay in diagnosis is the usual scenario in this syndrome, which makes patients more susceptible to complications. If patients with this triad are unresponsive to appropriate antibiotic treatment, they should be evaluated for abscess formation, a known complication of hip osteomyelitis [6]. If an abscess is present, surgical intervention would be indicated. As occurred in this case, reaching this point without appropriate management may lead to joint destruction with an emergent need for joint replacement surgery.

## Conclusion

Osteomyelitis, thrombophlebitis, and septic pulmonary embolism altogether represent a rare syndrome that usually presents in children with a history of trauma to long bones. Clinical evaluation of the associated DVT using duplex US only misleads the diagnosis. Thus, MRI evaluation for this group of patients is crucial and would prevent the unwilling complications of late diagnosis that include Sepsis, abscess formation, and joint destruction.

## Data Availability

Not applicable.

## References

[1] LePage AA, Hess EP, Schears RM (2008). Septic thrombophlebitis with acute osteomyelitis in adolescent children: a report of two cases and review of the literature. Int J Emerg Med.

[2] Lew DP, Waldvogel FA (2004). Osteomyelitis. Lancet.

[3] Gorenstein A (2000). The pivotal role of deep vein thrombophlebitis in the development of acute disseminated staphylococcal disease in children. Pediatrics.

[4] Tragiannidis A (2008). Septic pulmonary embolism due to *Staphylococcus aureus*. Pediatr Int.

[5] Okada F (2009). Clinical and pulmonary thin-section CT findings in acute *Klebsiella pneumoniae* pneumonia. Eur Radiol.

[6] Panteli M, Giannoudis PV (2016). Chronic osteomyelitis: what the surgeon needs to know. EFORT Open Rev.

